# DNA Molecule Classification Using Feature Primitives

**DOI:** 10.1186/1471-2105-7-S2-S15

**Published:** 2006-09-26

**Authors:** Raja Tanveer Iqbal, Matthew Landry, Stephen Winters-Hilt

**Affiliations:** 1 Department of Electrical Engineering and Computer Science, Tulane University, New Orleans, LA 70118, USA; 2 Department of Computer Science, University of New Orleans, New Orleans, LA, 70148, USA; 3 Research Institute for Children, New Orleans Children's Hospital, New Orleans, LA 70118, USA

## Abstract

**Background:**

We present a novel strategy for classification of DNA molecules using measurements from an alpha-Hemolysin channel detector. The proposed approach provides excellent classification performance for five different DNA hairpins that differ in only one base-pair. For multi-class DNA classification problems, practitioners usually adopt approaches that use decision trees consisting of binary classifiers. Finding the best tree topology requires exploring all possible tree topologies and is computationally prohibitive. We propose a computational framework based on feature primitives that eliminates the need of a decision tree of binary classifiers. In the first phase, we generate a pool of weak features from nanopore blockade current measurements by using HMM analysis, principal component analysis and various wavelet filters. In the next phase, feature selection is performed using AdaBoost. AdaBoost provides an ensemble of weak learners of various types learned from feature primitives.

**Results and Conclusion:**

We show that our technique, despite its inherent simplicity, provides a performance comparable to recent multi-class DNA molecule classification results. Unlike the approach presented by Winters-Hilt *et al*., where weaker data is dropped to obtain better classification, the proposed approach provides comparable classification accuracy without any need for rejection of weak data. A weakness of this approach, on the other hand, is the very "hands-on" tuning and feature selection that is required to obtain good generalization. Simply put, this method obtains a more informed set of features and provides better results for that reason. The strength of this approach appears to be in its ability to identify strong features, an area where further results are actively being sought.

## Background

**Figure 1:**
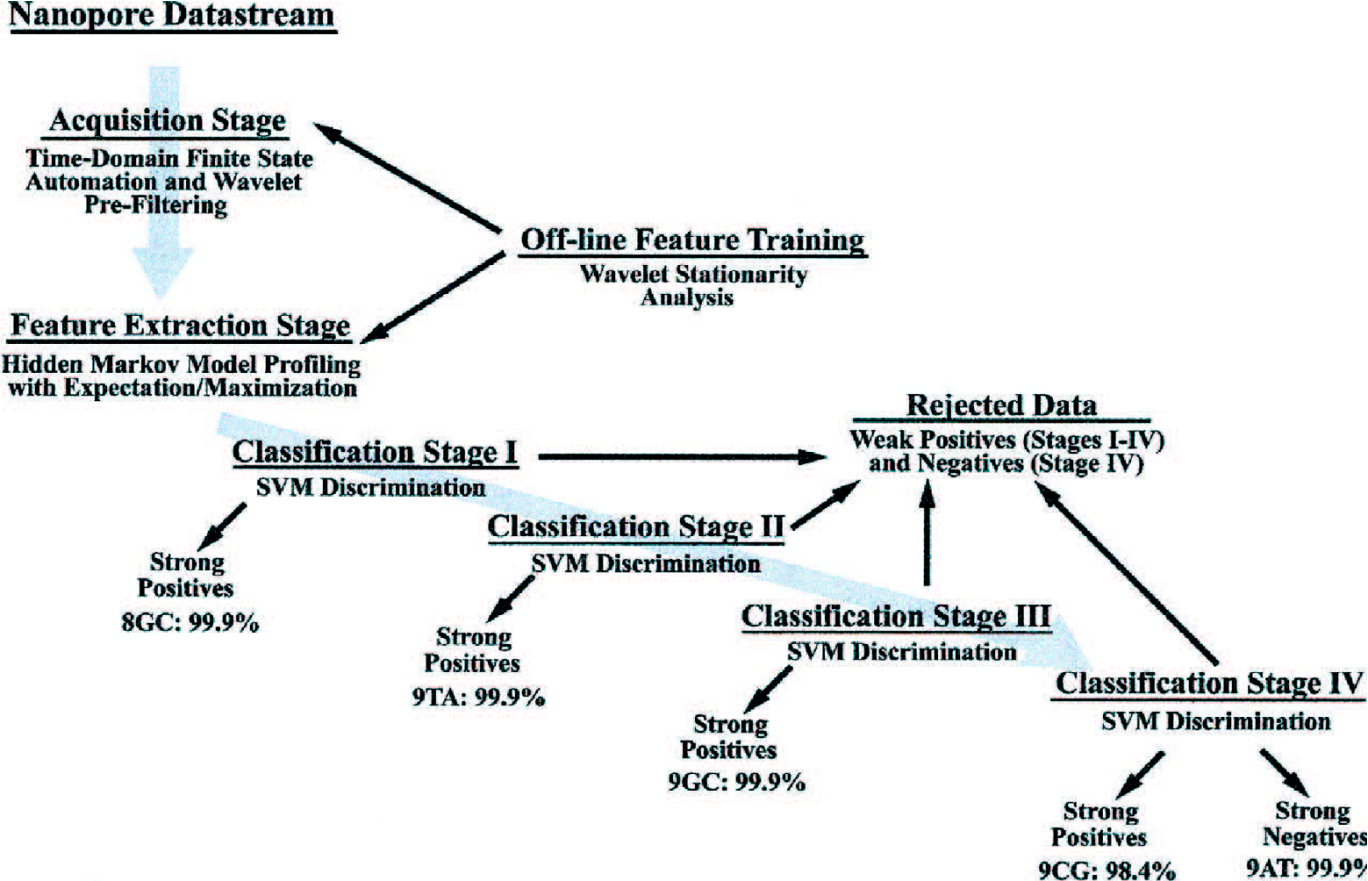
Classification technique adopted by Winters-Hilt et al. Source [6]

During the past decade, nanopore detectors have been shown to be helpful in DNA molecule classification [1-5]. The detectors relate ionic current blockade measurements from a nanometer-scale pore to single molecule translocation [1-3]. Alpha-Hemolysin channels provide inexpensive and reproducible nanopores due to their self assembling property in lipid bilayers. For DNA classification, the alpha-Hemolysin pore is optimal due to the fact that single-stranded DNA (ssDNA) translocates in alpha-Hemolysin pore whereas double-stranded DNA (dsDNA) does not. Instead it is held in a vestibule of the pore [5]. For DNA measurements using nanopores, an important milestone is the ability to rapidly identify individual bases or base-pairs in single DNA molecules. One end of double-stranded DNA (dsDNA) can be captured by the alpha-Hemolysin pore and held for an extended period of time [5]. Extensive characterization of the ionic current blockade associated with such an event is thus made possible. In [6], Winters-Hilt *et al*. use an SVM-based decision-tree to classify features vectors obtained from blockade current measurements from a nanopore detector. The DNA hairpins they choose differ only in one base pair. Their results show accuracies close to 99%. The classification strategy adopted by Winters-Hilt *et al*. is shown in Figure 1. In their technique, signal acquisition is performed using a time-domain, thresholding, Finite State Automaton. This is followed by adaptive pre-filtering using a wavelet-domain Finite State Automaton. Feature extraction on acquired channel blockades is done by Hidden Markov Model processing; and classification is done by Support Vector Machine (SVM). Figure 1 shows the optimal SVM architecture for classification of molecules 9CG, 9GC, 9TA, 9AT, and 8GC. The approach proposed by Winters-Hilt *et al*. provides excellent classification accuracy in classifying DNA hairpins that differ only in one base-pair. This approach requires a decision tree structure consisting of binary classifiers at each node. Each binary classifier assigns a class label to the input data or rejects the input data if the classification confidence is low. Strong negatives are handed to the next node (another binary classifier) in the decision tree. Although it can be automated (removing the expert from the problem application), the technique requires exploring all possible topologies of the SVM decision tree structure to be comprehensive. In practice, greatly reduced tree searches over linear topologies are indicated in [6]. Even with the linear tree exploration, however, training the decision tree can be time consuming and computationally expensive. We propose a technique that replaces the SVM decision tree structure proposed in [6] with a classification frame work based on boosting. The proposed framework begins with the same features as used by Winters-Hilt *et al*. and then generates more features from the existing set of features by applying wavelet filters and principal component analysis on the original features (which partly recovers transition probability information lost in the feature compression used in [6]). AdaBoost is used to perform selection of weak classifiers learned from the enhanced feature set consisting of the original and derivative features. An ensemble is obtained that consists of a weighted vote of the weak learners chosen by AdaBoost.

### Nanopore Detectors: Experimental Setup

**Figure 2:**
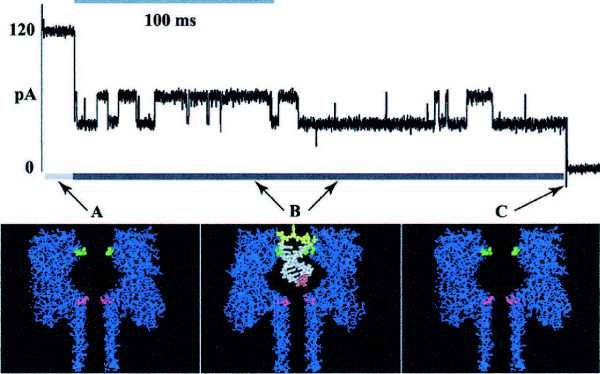
Examination of DNA duplex ends using a voltage-pulse routine. Source [6]

Each experiment is conducted using one alpha-Hemolysin channel inserted into a diphytanoyl-phosphatidylcholine/hexadecane bilayer as shown in Figure 2, where the bilayer is formed across a 20-micron diameter horizontal Teflon aperture [5]. The bilayer separates two 70 μ*L *chambers containing 1.0 M KCl buffered at pH 8.0 (10 mM HEPES/KOH). A completed bilayer between the chambers is indicated by the lack of ionic current flow when a voltage is applied across the bilayer (using Ag-AgCl electrodes). Once the bilayer is in place, a dilute solution of alpha-Hemolysin (monomer) is added to the *cis *chamber. Self-assembly of the alpha-Hemolysin heptamer and insertion into the bilayer results in a stable, highly reproducible, nanometer-scale channel with a steady current of 120 pA under an applied potential of 120 mV at 23C (using a Peltier device). Once one channel is formed, further pores are prevented from forming by thoroughly perfusing the *cis *chamber with buffer. Molecular blockade signals are then observed by mixing analytes into the *cis *chamber.

The nine base-pair hairpin molecules examined share an eight base-pair hairpin core sequence, to which one of the four permutations of Watson-Crick base-pairs that may exist at the blunt end terminus are attached, i.e. 5'-GC-3', 5'-CG-3', 5'-TA-3', and 5'-AT-3'. These are denoted by 9GC, 9CG, 9TA, and 9AT. The sequence of the 9CG hairpin is 5'-CTTCGAACGTTTTCGTTCGAAG-3'. The base-pairing region is underlined. An eight base-pair DNA hairpin with a 5'-GC-3' terminus was also tested. This control molecule is denoted by 8GC. The DNA oligonucleotides were synthesized using an ABI 392 Synthesizer, purified by PAGE, and stored at -70C in TE buffer. The prediction that each hairpin would adopt one base-paired structure was tested and confirmed using the DNA mfold server [7].

**Figure 3:**
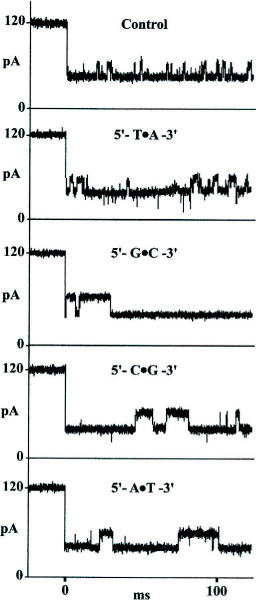
Typical blockade signatures for each of the five classes of DNA hairpins. Source [6]

**Figure 4:**
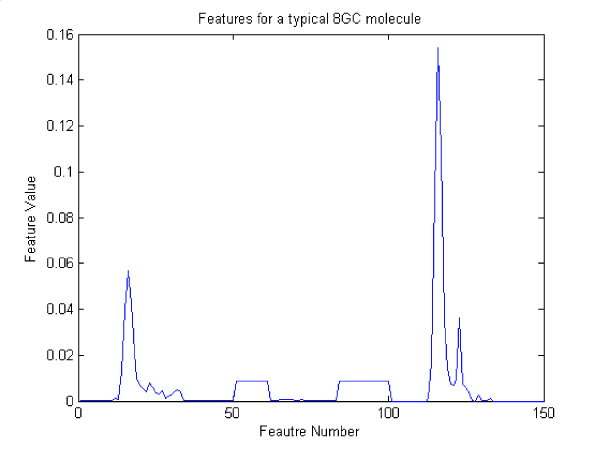
Features for a typical 8GC type molecule.

**Figure 5:**
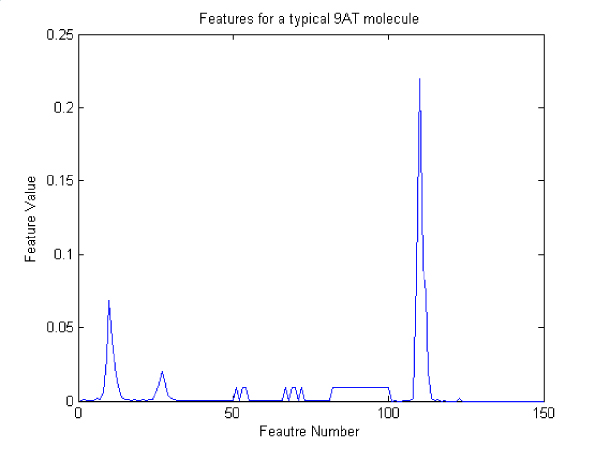
Features for a typical 9AT type molecule.

**Figure 6:**
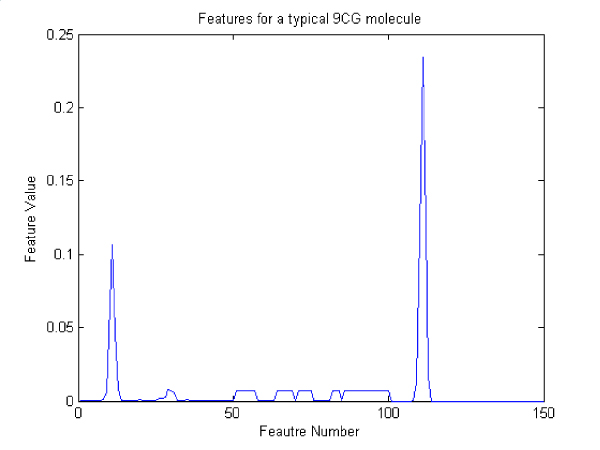
Features for a typical 9CG type molecule.

**Figure 7:**
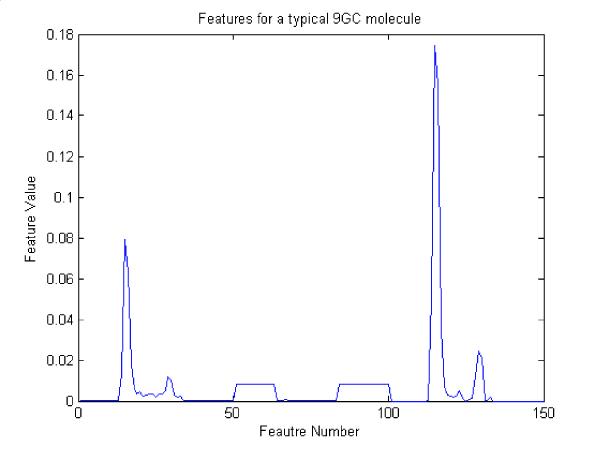
Features for a typical 9GC type molecule.

**Figure 8:**
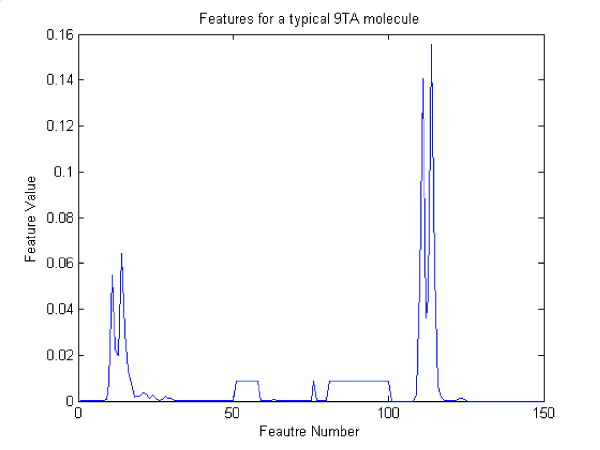
Features for a typical 9TA type molecule.

In Figure 2, an observation cycle for a 9GC hairpin blockade event is shown. At the start of each voltage cycle the voltage across the pore is reset to 0 mV. A potential difference of 120 mV (*trans *side positive) is then applied for 250 ms, initially resulting in an open channel current of 120 pA (image labeled A in Figure 2, with arrow indicating the open channel region of the current trace). In time, duplex DNA is pulled into the pore by the applied potential causing an abrupt current decrease (image B, with arrows and solid bar delineating region of blockade signal). After the 250 ms forward bias, the potential is briefly reversed (-40 mV, *trans *side) then set at 0 mV for 50 ms which clears the pore (image C, with arrow indicating the voltage reversal spike). The cycle is then repeated to examine the next molecule. Only the first 100 ms of blockade signal is used to identify each current signature. In the diagrams, the stick figure in blue is a two-dimensional section of the alpha-Hemolysin pore derived from x-ray crystallographic data [8]. A ring of lysines that circumscribe a 1.5 nm limiting aperture of the channel pore is highlighted in red. A ring of threonines that circumscribe the narrowest 2.3 nm diameter section of the pore mouth is highlighted in green. In our working model, the four dT hairpin loop (yellow) is perched on this narrow ring of threonines, suspending the duplex stem in the pore vestibule [5]. The terminal base-pair (brown) dangles near the limiting aperture. The structure of the 9 bp hairpin shown here is rendered to scale using WebLab ViewerPro. Once the blockade current measurements are obtained, features are obtained using time domain finite state automata and wavelet pre-filtering followed by HMM profiling with expectation maximization. The feature extraction process can be found in [6]. Whenever we use the term HMM projections in the remaining part of this report, it would refer to the features extracted using the method explained in this section. The process of feature extraction can be found in a greater detail in [6]. Typical blockade signatures for each of the five classes of DNA hairpins are shown in Figure 3. The nine base-pair hairpins differ in only their terminal base-pairs. The variants are chosen to include the two possible Watson-Crick base-pairs and the two possible orientations of those base-pairs at the duplex ends. The core 8 bp stem and 4dT loop are identical with the primary sequence 5'-TTCGAACGTTTTCGTTCGAA-3'. Signature HMM Projections for the five DNA hairpins (8GC, 9AT, 9CG, 9GC, 9TA) are shown in Figure 4, Figure 5, Figure 6, Figure 7 and Figure 8.

### AdaBoost: An Overview

AdaBoost [9-11] is an iterative scheme to obtain a weighted ensemble of weak learners. The basic idea is that one can combine rules of thumb to form an ensemble whose joint decision rule has good performance on the training set. Successive component classifiers are trained on a subset of the training data that is most informative. AdaBoost learns a sequence of weak classifiers and then boosts them by a linear combination into a single strong classifier. The input to the algorithm is a training set {(x_1_, y_1_), ..., (x_*N*_, y_*N*_)} where y_i _∈ Y = {-1, +1} is the correct label of instance x_i _∈ X and *N *is the number of training examples in the data set. A weak learning algorithm is repeatedly called in a series of rounds *t *= 1, ..., *T *with different weights distributions D_*t *_on the training data. This set of weights associated with the training data at each round *t *is denoted by D_*t*_(i). In general, sampling weights associated with each example are initially set equal, i.e. a uniform sampling distribution is assumed. For the *t*^th ^iteration, a classifier is learned from the training examples and the classifier with error ε_*t *_≤ 0.5 is selected. In each iteration, the weights of misclassified examples are increased which results in these examples getting more attention in subsequent iterations. AdaBoost is outlined in Algorithm 1 below. It is interesting to note that α_*t *_measures the importance assigned to the hypothesis *h*_*t *_and it gets larger as the training error ε_*t *_gets smaller. The final classification decision *H *of a test point *x *is a weighted majority vote of the weak hypotheses.

**Algorithm 1. **The AdaBoost algorithm

**Input: ***S *= {(x_1_, y_1_), ..., (x_N_, y_N_)} where *x*_*i *_∈ X and *y*_*i *_∈ Y = {-1, +1}

**Initialization: ***D*_1_(*i*) = 1/*N*, for all *i *= 1, ..., *N*

**For ***t *= 1 *to T ***do**

1. Train weak learners with respect to the weighted sample set {*S,D*_*t*_} and obtain hypothesis *h*_*t *_: *X *→ *Y*.

2. Obtain the error rates ε_*t *_of *h*_*t *_over the distribution D_*t *_such that

ε_*t *_=  [*h*_*t*_(x_*i*_) ≠ *y*_*i*_].

3. Set α_*t *_= 1/2 *ln*(1-ε_*t*_/ε_*t*_)

4. Update the weights: D_*t*+1_(*i*) = (D_*t*_(*i*)/Z_t_) , where Z_t _is the normalizing factor such that D_*t*+1_(*i*) is a distribution.

5. Break if ε_*t *_= 0 or ε_*t*_≥ 1/2.

end

**Output: ***H*(*x*) = *sign*(Σ^T^_t = 1_α_*t *_*h*_*t*_(x_i_))

### DNA Molecule Classification Using Boosted Naive Bayes

Given *n *classes and an input **x**, naive Bayes assigns to **x **the class label ω_*i *_for class *i *for which the posterior probability given by the following expression is maximum:

*p*(ω_*i *_| **x**) = *p*(**x **| ω_*i*_)*p*(ω_*i*_)/Σ^n^_j = 1 _*p*(**x **| ω_*j*_)*p*(ω_*j*_).

The probability *p*(ω_*i*_) is the prior probability that represents the fraction of examples in the dataset that belong to class ω_*i *_and *n *in the total number of class labels that are possible. The probability *p*(**x **| ω_*i*_) is computed by making the assumption that the features in the dataset are independent and hence the probability *p*(**x **| ω_*i*_) is given by

*p*(**x **| ω_*i*_) = ∏^m^_j = 1 _*p*(x_j _| ω_*i*_),

where *m *is the total number of features. This is a very strong assumption but has been shown to work in practice. The label class label predicted by the naive Bayes classifiers is the one for which the *p*(ω_*i*_) is maximum. For example, for a two class problem we have *n *= 2 and hence if *p*(ω_1 _| **x**) *> p*(ω_2 _| **x**) then label is predicted to be label '1' and is predicted label '2' otherwise. An attempt to obtain classifiers in one against rest and all pairs settings using only the HMM features was made as a first step. After several rounds of boosting, no weak learner with an accuracy greater than 50% was found. This can be attributed to the fact that some features in the HMM projections are noisy which are affecting the posterior probability and hence no weak learner is obtained. We then perform principal component analysis (PCA) [12] on the HMM projection data. We noticed that 90% of the information is contained in the first 50 principal components. We hence use only first 50 principal components as our new feature set. Naive Bayes classifiers are used once again as weak learners for AdaBoost in one against rest and all pairs settings. The ensembles obtained by AdaBoost for each case provided reasonable accuracy in one against all and all pairs settings. The classification results obtained are summarized in Table 1 and Table 2.

In order to obtain a single classifier for classifying all five molecules a decision tree structure is used, where each of the nodes is a binary classifier which classifies the input into two groups. This process is repeated until a single class label for the input has been found. As discussed in earlier sections, this approach is computationally expensive as choosing the right topology for the decision tree structure would require empirically evaluating all possible topologies (for the datasets examined in [6], however, linear trees were found to be optimal with drop of weak data). In the following section we discuss a framework that eliminates the need for a decision tree structure for multiclass classification.

### DNA Molecule Classification Using Boosting Over Stumps

**Figure 9:**
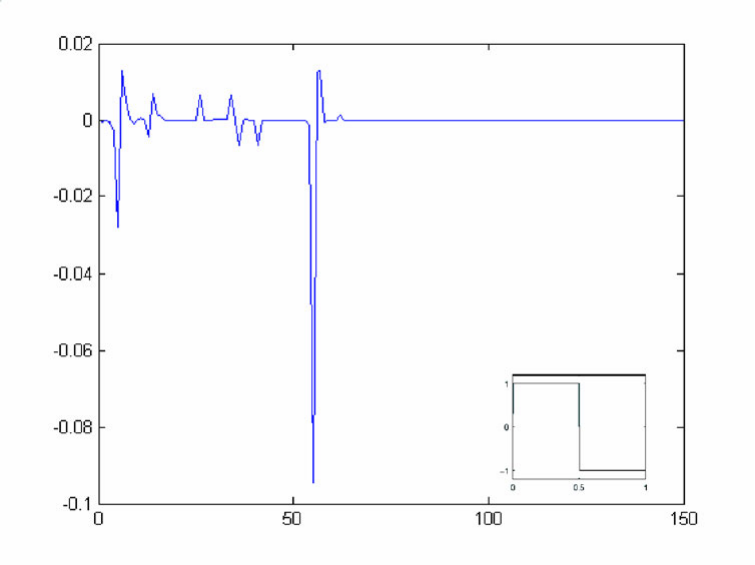
Features obtained for an 8GC hairpin after applying a Haar wavelet filter.

**Figure 10:**
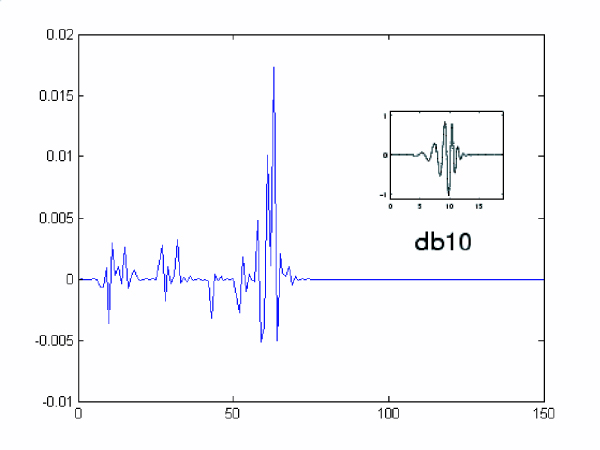
Features obtained for an 8GC hairpin after applying a 10*th *order Daubechies wavelet filter.

To obtain a single multiclass learner, the boosting approach proposed in the previous section was modified. We generate more features from the HMM projections hoping that the new features will be able to capture additional 'structure' in the original dataset. We applied Haar, Daubechies and Symlets wavelet filters of different orders on the HMM projections and used them to enhance the existing feature set. Figure 9 and Figure 10 show the features obtained as a result of applying Haar and Daubechies wavelet filters. The weak learners are then obtained using density estimation over individual features. Typically AdaBoost is used to perform classification for binary classification problems. To perform classification of five classes of molecules the AdaBoost approach was modified. For each class label ω_*i*_, the probability *p*(ω_*i *_| **x**) is computed using the Bayes formula given above, and the label belonging to the class corresponding to the highest posterior probability is considered the predicted label. It should be noted that **x **is no longer a vector of features. Instead it is just an individual feature, and as a result there is not need to evaluate *p*(**x **| ω_*i*_) as a product of various probabilities.

## Results and Discussion

We applied several rounds of AdaBoost on data sets consisting of following feature sets

• Data set I: HMM Projections

• Data set II: Data set I enhanced with first 50 principal components obtained from HMM projections, approximation and detail coefficients obtained using a haar filter

• Data set III: Data set II enhanced with approximation and detail coefficients obtained using a second and tenth order Daubechies wavelet filter

• Data set IV: Data set III enhanced with approximation and detail coefficients obtained using a second and tenth order Symlets wavelet filter

**Figure 11:**
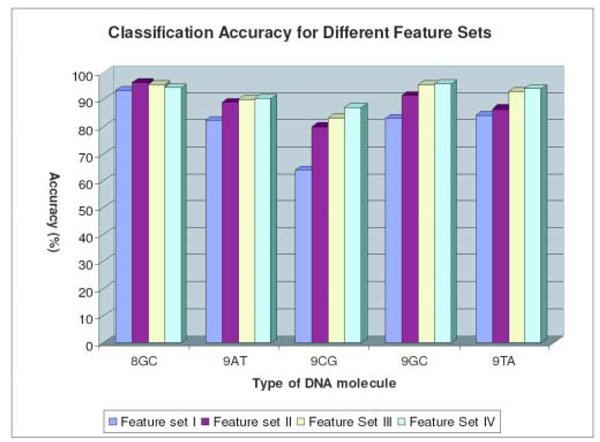
Classification Results for four different datasets used in boosting with stumps approach.

In each case number of rounds of boosting were equal to the total number of features available in the data set. The classification results are shown in Figure 11. It can be seen that the overall classification performance improves as more feature types are added to the dataset. In Figure 11, when only HMM features were used, the classification accuracy for the 8GC, 9AT, 9CG, 9GC, and 9TA molecules was 93.3%, 82.3%, 64.1%, 83.1%, and 84.3% respectively. This performance is remarkable, considering the fact that boosted naive Bayes was not even able to obtain a weak learner using HMM features. This handicap of naive Bayes can be attributed to the independence assumption in computing the joint probabilities of features. In the proposed approach, weak learners are obtained using individual features (feature primitives) and not a group of features. It should be noted, however, that the Daubechies and Symlet filters re-couple the components. As a result of the use of primitives in the set of learners, one poor feature cannot affect a good feature just because they are both being used to learn a weak classifier at the same time. The classification performance as more types of features are added can be seen in Figure 11.

## Figures and Tables

**Table 1:** Results of one against rest approach on principal components obtained from the HMM projections.

Class1	Class2	Sensitivity	Specificity
8GC	9AT,9CG,9GC,9TA	0.9549	0.9758
9AT	8GC,9CG,9GC,9TA	0.9295	0.9161
9CG	8GC,9AT,9GC,9TA	0.8143	0.9434
9GC	8GC,9AT,9CG,9TA	0.8156	0.9452
9TA	8GC,9AT,9CG,9GC	0.8501	0.9902

**Table 2:** Results using all pairs approach on principal components obtained from the HMM projections.

	8GC	9AT	9CG	9GC	9TA
8GC	x	Sens = 97.30 Spec = 98.00	Sens = 97.30 Spec = 98.25	Sens = 98.85 Spec = 97.95	Sens = 97.15 Spec = 98.15
8GC	x	x	Sens = 96.50 Spec = 98.50	Sens = 99.25 Spec = 98.75	Sens = 96.40 Spec = 94.30
8GC	x	x	x	Sens = 98.20 Spec = 93.80	Sens = 96.40 Spec = 94.30
8GC	x	x	x	x	Sens = 95.70 Spec = 95.15

## References

[B1] Akeson M, Branton D, Kasianowicz J, Brandin E, Deamer DW (1999). Microsecond time-scale discrimination among polycytidilic acid, polyadenylic acid and polyuridylic acid as homopolymers or as segments within single RNA molecules. Biophysical Journal.

[B2] Kasianowicz J, Brandin E, Deamer DW (1996). Characterization of individual polynucleotide molecules using a membrane channel. Proceedings of National Academy of Sciences.

[B3] Meller A, Nivon L, Brandin E, Golovchenko J, Branton D (2000). Rapid nanopore discrimination between single polynucleotide molecules. Proceedings of National Academy of Sciences.

[B4] Meller A, Nivon L, Branton D (2001). Voltage-driven DNA translocations through a nanopore. Physical Review Letters.

[B5] Vercoutere W, Winters-Hilt S, Olsen H, Deamer D, Haussler D, Akeson M (2001). Rapid discrimination among individual DNA hairpin molecules at single-nucleotide resolution using an ion channel. Nature Biotechnology.

[B6] Winters-Hilt S, Vercoutere W, DeGuzman VS, Deamer D, Akeson M, Haussler D (2003). Highly Accurate Classification of Watson-Crick Base-pairs on Termini of Single DNA Molecules. Biophysical Journal.

[B7] SantaLucia J (1998). A unified view of polymer, dumbbell, and oligonucleotide DNA nearest-neighbor thermodynamics. Proceedings of National Academy of Sciences.

[B8] Song L, Hobaugh M, Shustak C, Cheley S, Bayley H, Gouaux JE (1996). Structure of staphylococcal alpha-Hemolysin, a heptameric transmembrane pore. Science.

[B9] Freund Y, Schapire R (1997). A decision-theoretic generalization of on-line learning and an application to boosting. Journal of Computer and System Sciences.

[B10] Fruend Y, Schapire RE, Bartlett P, Lee WS (1998). Boosting the margin. a new explanation for the effectiveness of voting methods. Annals of Statistics.

[B11] Schapire RE, Singer Y (1999). Improved Boosting Using Confidence-rated Predictions. Machine Learning.

[B12] Duda R, Hart P, Stork D (2001). Pattern Classification.

